# Generation of a Reassortant Influenza A Subtype H3N2 Virus Expressing *Gaussia* Luciferase

**DOI:** 10.3390/v11070665

**Published:** 2019-07-20

**Authors:** Lin Wang, Qinghua Cui, Xiujuan Zhao, Ping Li, Yanyan Wang, Lijun Rong, Ruikun Du

**Affiliations:** 1 College of Pharmacy, Shandong University of Traditional Chinese Medicine, Jinan 250355, China; 2 Shandong Provincial Collaborative Innovation Center for Antiviral Traditional Chinese Medicine, Jinan 250355, China; 3 Qingdao Academy of Chinese Medicinal Sciences, Shandong University of Traditional Chinese Medicine, Qingdao 266122, China; 4 Department of Microbiology and Immunology, College of Medicine, University of Illinois at Chicago, Chicago, IL 60612, USA

**Keywords:** influenza A virus, subtype H3N2, reassortant, reporter virus

## Abstract

Reporter influenza A viruses (IAVs) carrying fluorescent or luminescent genes provide a powerful tool for both basic and translational research. Most reporter IAVs are based on the backbone of either subtype H1N1 viruses, A/Puerto Rico/8/1934 (PR8) or A/WSN/1933, but no reporter subtype H3N2 virus is currently available to our knowledge. Since the IAV subtype H3N2 co-circulates with H1N1 among humans causing annual epidemics, a reporter influenza A subtype H3N2 virus would be highly valuable. In this study, the segments of A/Wyoming/3/03 (NY, H3N2) virus encoding hemagglutinin and neuraminidase, respectively, were reassorted with the six internal genes of PR8 where the NS gene was fused with a *Gaussia* luciferase (Gluc) gene. Using reverse genetics, NY-r19-Gluc, a replication competent reassortant influenza A subtype H3N2 virus expressing reporter Gluc was successfully generated. This reporter virus is stable during replication in Madin-Darby canine kidney (MDCK) cells, and preliminary studies demonstrated it as a useful tool to evaluate antivirals. In addition, NY-r19-Gluc virus will be a powerful tool in other studies including the application of diagnostic and therapeutic antibodies as well as the evaluation of novel vaccines.

## 1. Introduction

The influenza A virus (IAV) is a major cause of respiratory illness in humans, accounting for up to 650,000 annual deaths worldwide [1]. The IAV belongs to the family *Orthomyxoviridae* of enveloped viruses, and the genome consists of 8 negative-sense, single-stranded viral RNA (vRNA) segments, coding for at least 11 proteins [2]. Hemagglutinin (HA) and neuraminidase (NA) are the major surface glycoproteins, based on which IAVs can be classified into different subtypes. There are 16 subtypes of HA (H1–H16) and 9 subtypes of NA (N1–N9) identified in wild birds [3]. In addition, two new subtypes (H17N10 and H18N11) have recently been isolated in bats [4,5]. Currently, IAV subtypes H1N1 and H3N2 as well as two influenza B virus strains of Yamagata and Victoria lineages co-circulate in humans [6], while other subtypes such as H5N1 and H7N9 can gain the ability to infect humans occasionally [7,8].

The HA protein is the most important antigen in IAVs and plays a critical role in receptor binding and membrane fusion during virus entry, being an attractive target for developing vaccines and anti-IAV drugs [9,10]. Based on sequence comparisons and structural characteristics, the HA subtypes are further divided into five clades that fall into two groups. Group 1 consists of subtypes H1, H2, H5, H6, H8, H9, H11, H12, H13, H16, H17, and H18, and Group 2 is composed of subtypes H3, H4, H14, H7, H15, and H10 [11]. The genetic similarity of HA amino acid sequences between the subtypes within each group is about 60–74%, while the similarity between different groups is only 40–44% [10]. Seasonal influenza vaccines are prepared in advance according to the recommendations issued by WHO, containing two IAV strains (H1N1 and H3N2) and one (trivalent influenza vaccine) or two (quadrivalent influenza vaccine) influenza B virus (IBV) strains. These vaccines usually become less effective when the vaccine virus strains do not match the circulating strain(s), particularly in the context of an influenza pandemic [12]. Several compounds have been shown to block virus infection as HA inhibitors, most of which, however, showed subtype-specificity. Inhibitors for Group 1 HAs include CL-61917, CL-385319, and CL-62554 [13], Stachyflin [14], BMY-27709 [15,16], LY-180299 [17], RO5464466 and RO5487624 [18], MBX2546 [19], and FA-583 and FA-617 [20], while TBHQ [21,22], and S19 and C22 [23] are currently known inhibitors targeting group 2 HAs. Arbidol has been shown to have inhibitory effects against HAs of both group 1 and group 2. However, Arbidol is likely not an IAV-specific antiviral, since it is effective against a number of enveloped and non-enveloped viruses [24].

Reverse genetics is a useful tool for the development of novel vaccines and antiviral drugs. For example, escape mutants can be investigated by reverse genetic technique, providing clues to the binding site of a target protein and mechanism of action for an antiviral [20]. In addition, replication competent recombinant reporter IAVs that express fluorescent proteins or luciferases provide a simple and robust approach for antiviral studies [25,26,27,28]. However, most currently available reporter IAVs are constructed with either A/Puerto Rico/8/1934 (H1N1; PR8) or A/WSN/1933 (H1N1) as the backbone (reviewed in Reference [29]). For other subtypes, to our knowledge, only reporter IAVs of H5N1 and H7N9 have been generated [30,31]. In spite of their clinical importance, reporter IAVs of subtype H3N2 have not been reported. Here we report the successful generation of a reporter reassortant influenza A subtype H3N2 virus carrying the *Gaussia* luciferase (Gluc) gene.

## 2. Materials and Methods

### 2.1. Cells, Compounds, and Viruses

Human embryonic kidney cell line 293T and Madin-Darby canine kidney (MDCK) epithelial cells were obtained from Dr. Fei Deng (Wuhan Institute of Virology, CAS, China) and grown in Dulbecco’s modified Eagle’s medium (DMEM; Cellgro, Manassas, VA, USA) supplemented with fetal bovine serum (10%; Gibco, Carlsbad, CA, USA), penicillin and streptomycin (1000 units/mL and 100 µg/mL, respectively; Invitrogen, Carlsbad, CA, USA). Opti-MEM containing 2 µg/mL N-tosyl-L-phenylalanine chloromethyl ketone (TPCK)–trypsin (Sigma-Aldrich, St. Louis, MO, USA) was used to perform infections. All cells were grown at 37 °C in 5% CO_2_.

Compounds tert-Butylhydroquinone (TBHQ) and arbidol were purchased from MedChemExpress (Monmouth Junction, NJ, USA).

Reporter virus PR8-NS1-Gluc (referred to as A/PR8-Gluc [H1N1] here) and parental virus A/PR8 stocks were produced as previously described [28].

### 2.2. Plasmids

The rescue plasmids for the internal segments of the PR8 backbone, including pDZ-PR8/PB2, -PR8/PB1, pDZ-PR8/PA, -PR8/NP, -PR8/M, -PR8/NS, and -PR8/NS-Gluc were used as previously described [28]. The rescue plasmids of A/Wyoming/3/03 (NY, H3N2), including pDZ-NY/PB2, -NY/PB1, -NY/PA, -NY/HA, -NY/NP, -NY/NA, -NY/M, and -NY/NS were kindly provided by Adolfo Garcia-Sastre (Mt Sinai School of Medicine, New York, NY, USA).

The plasmids pDZ-NY/NS-Gluc were constructed as follows:

(i) Construction of pDZ-NY/NS-SAnull in which the splicing acceptor site was disrupted

The NY/NS-SAnull-left and NY/NS-SAnull-right fragments were amplified from pDZ-NY/NS using primer pairs 5′-INFU-NCR-NYNS1-F/3′-SDM-NYNS-SAnull-R and 5′-SDM-NYNS-SAnull-F/3′-INFU-NYNEP-NCR-R respectively. Using an In-Fusion cloning kit, the NY/NS-SAnull-left and NY/NS-SAnull-right fragments were cloned into *Sap* I linearized pDZ plasmid, and the resulting plasmid carries what is herein referred to as the NS ‘Splice Acceptor null’ (SA-null) gene.

(ii) Construction of pDZ-NY/NS-Gluc

Using pDZ-NY/NS-SAnull as a template, fragments NY/NS-Gluc-1 and NY/NS-Gluc-3 were amplified by primer pairs 5′-INFU-NCR-NYNS1-F/3′-INFU-NYNS1-nostop-R and 5′-INFU-NYNEP-F/3′-INFU-NYNEP-NCR-R respectively.

NY/NS-Gluc-2 was amplified from pDZ-PR8/NS-Gluc using primers 5′-INFU-Gluc-F and 3′-INFU-Gluc-2A-R.

Using an In-Fusion cloning kit, the NY/NS-Gluc-1, NY/NS-Gluc-2 and NY/NS-Gluc-3 fragments were cloned into *Sap* I linearized pDZ plasmid.

All primers used in this process are listed in Table 1.

### 2.3. Generation of Reporter IAV of H3N2

As described previously, wild-type A/NY (H3N2) was generated by using the reverse genetics system with indicated rescue plasmids [32]. Briefly, eight pDZ plasmids (0.5 μg each) representing the eight segments of the IAV genome were transfected into 293T/MDCK cocultures using Lipofectamine 2000 (Invitrogen, Carlsbad, CA, USA). After 48 h the virus was harvested from the supernatant.

In order to generate reporter IAV H3N2, three strategies were employed respectively.

First, pDZ-NY/NS-Gluc instead of pDZ-NY/NS was used to generate a recombinant virus in which the NS gene of the NY virus was fused with the reporter gene *Gaussia* luciferase, which is the same as the strategy used to construct PR8/NS-Gluc previously described [28]. The recombinant virus was designated as NY-Gluc accordingly.

Second, the NS segment of the NY virus was replaced with PR8/NS-Gluc, and the virus was designated as NY-r18-Gluc.

Third, the six segments other than HA and NA of the NY virus were replaced with those of PR8-Gluc, and the virus was designated as NY-r19-Gluc. In parallel, a reassortant NY-r19 virus was generated by replacing the six internal segments of NY with those of wild-type PR8 virus.

The TCID_50_ values were determined using MDCK cells and the titer was calculated by the Reed–Muench method [33].

### 2.4. Virus Propagation

The viruses of NY, NY-Gluc, NY-r18-Gluc, NY-r19, and NY-r19-Gluc were propagated in MDCK cells. MDCK cells of 80% confluence grown in 10 cm dishes were infected with the indicated viruses at a multiplicity of infection (MOI) of 0.01 TCID_50_ per cell. After incubation at 37 °C for 1 h, the cells were washed and fresh medium was added. The viruses were harvested respectively when most cells showed the cytopathic effect (CPE) and titrated for TCID_50_ values.

In order to test the genetic stability of reporter IAV NY-r19-Gluc, the virus was serially passaged in MDCK cells eight times, labeled as P1 to P8 accordingly.

### 2.5. Multicycle Replication Assay

A multicycle replication assay was performed to compare the replication properties of the indicated viruses in vitro. Briefly, MDCK cells were seeded into 24-well plates 1 day before inoculation with the indicated viruses at a MOI of 0.01. After incubation at 37 °C for 1 h, the cells were washed, followed by addition of fresh medium. At various time points, aliquots were removed for determination of Gluc activity and viral titers.

### 2.6. Gaussia Luciferase Assay

The *Gaussia* luciferase assay was performed as previously described [28]. Briefly, 50 µL of viral culture medium was examined using the BioLux Gaussia Luciferase Assay Kit (NEB, Ipswich, MA, USA), and the relative light units (RLU) were detected using SIRIUS L Luminometer (Titerteck Berthold, Bad Wildbad, Germany).

### 2.7. Antiviral Determination

MDCK cells grown in 24-well plates were infected with NY-r19-Gluc virus at a MOI of 0.1. After 1 h of incubation at 37 °C, the cells were washed, and fresh Opti-MEM containing 2 µg/mL TPCK-trypsin was added. For antiviral determination, indicated concentrations of TBHQ or arbidol were present all through the process. At around 72 h post-infection (p.i.), aliquots were removed for the luciferase assay.

## 3. Results

### 3.1. Reporter Influenza A Subtype H3N2 Virus with a Gluc Gene Fused to NS1 Loses the Reporter Gene Rapidly

**Figure 1:**
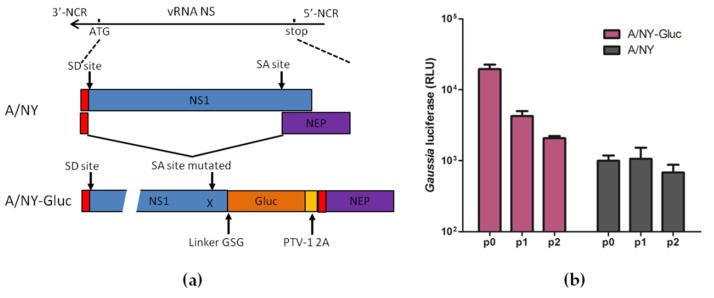
Generation of reporter influenza A subtype H3N2 virus by fusing *Gluc* gene to NS segment. (**a**) Engineering of Gluc reporter gene in the genome of influenza A NY (H3N2) virus. The porcine teschovirus–1 2A (PTV-1 2A) site allows reporter virus A/NY-Gluc to express both NS1-Gluc and NEP proteins from a single mRNA. SD/SA: Splice donor/acceptor sites. (**b**) Madin-Darby canine kidney (MDCK) cells were infected with WT NY or with NY-Gluc from serial passage experiments in MDCK cells (passages 0 to 2) at a MOI of 0.01. At 60 h post-infection (p.i.) luciferase assays were performed.

Previously, a reporter influenza A subtype H1N1 virus PR8-Gluc was successfully generated by fusing a Gluc gene to the NS1 open reading frame [28]. In this study, a similar strategy was initially employed to generate the reporter influenza A subtype H3N2 virus, NY-Gluc (Figure 1a). However, as the recombinant virus was passaged in MDCK cells, the expression level of the reporter Gluc decreased rapidly, and only negligible Gluc was detectable after two passages (Figure 1b). This result suggested that the NY-Gluc is genetically unstable in vitro.

### 3.2. Reporter Influenza A Subtype H3N2 Virus Carrying PR8/NS-Gluc Segment Induces CPE Alteration

To generate the reporter influenza A subtype H3N2 virus, another strategy was explored based on previous work by Fukuyama et al. [30]. They successfully generated a reporter IAV expressing fluorescent Venus based on A/Vietnam/1203/2004 (VN1203; H5N1) [30]. In this strategy, the PR8 NS gene is used to express NS1-Venus chimeric protein since the NS gene contributes little to the pathogenicity of VN1203 in mice [34].

**Figure 2:**
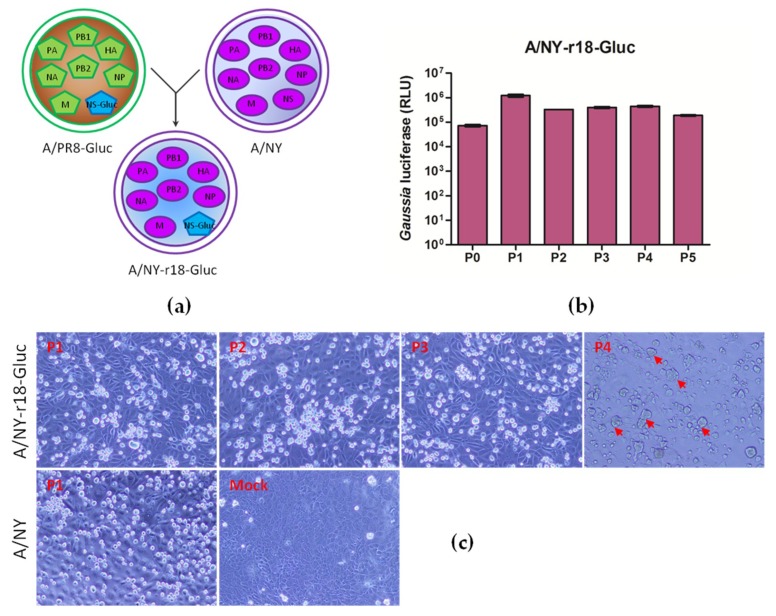
Generation of reporter influenza A subtype H3N2 virus by using segment PR8/NS-Gluc, which expresses NS1-Gluc chimeric protein. (**a**) Diagram of reassorment for generating reporter A/NY-r18-Gluc virus. (**b**) MDCK cells were infected with NY-r18-Gluc virus from serial passage experiments in MDCK cells (passages 0 to 5) at a MOI of 0.01. At 60 h p.i. luciferase assays were performed. (**c**) MDCK cells were mock-infected or infected with NY virus and NY-r18-Gluc virus of indicated passages respectively at a MOI of 1. Cytopathic effects were observed at 60 h p.i. using Olympus inverted phase contrast microscope IX73 (magnification 200×). Arrows indicate the multinucleated syncytia induced by NY-r18-Gluc infection.

As shown in Figure 2a, a reassortant virus containing PR8 NS-Gluc segment derived from a recombinant virus PR8-Gluc and the other seven segments of wildtype NY virus was successfully rescued using reverse genetics. The virus was designated as NY-r18-Gluc. Unlike the NY-Gluc virus, the NY-r18-Gluc virus could express Gluc protein at a high level, and no obvious decrease of Gluc expression was observed after serial passage of the viruses, suggesting that the reporter Gluc gene is functionally maintained (Figure 2b).

However, an altered CPE upon NY-r18-Gluc infection into MDCK cells was observed. As shown in Figure 2c, the infection of NY-r18-Gluc of passages 1 to 3 respectively caused cells to become round and detached from the culture plates, which is similar to the CPE induced by wildtype NY virus infection. However, after four passages, the NY-r18-Gluc infected cells became fibrous, and formed numbers of multinucleated syncytia (Figure 2c). This phenomenon implies that NY-r18-Gluc has evolved during passaging in MDCK cells, and the precise mutations and underlying mechanisms remain to be investigated, leaving the reporter virus with limited importance at present.

### 3.3. A 6:2 Genetic Reassortant Reporter Influenza A Subtype H3N2 Virus is Genetically Stable

**Figure 3:**
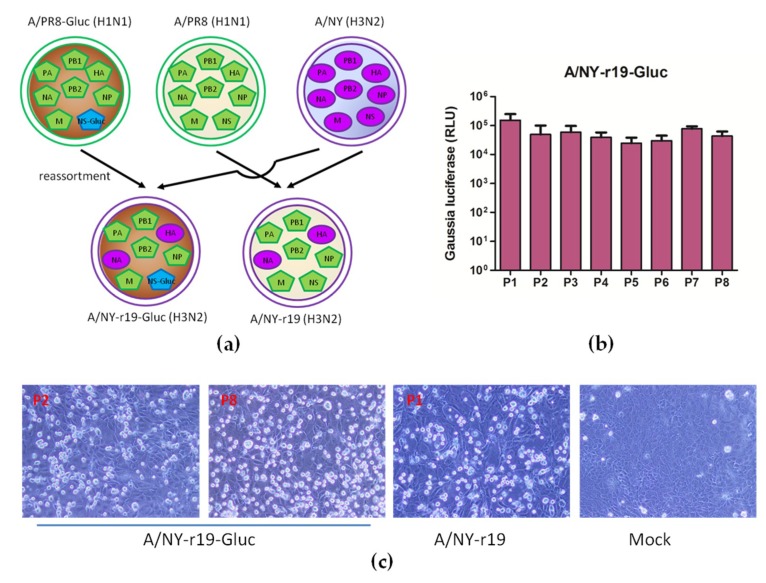
Generation of reporter influenza A subtype H3N2 virus encoding the HA and NA genes of wildtype NY virus, in a backbone comprising the six internal genes of PR8. (**a**) Diagram of reassorment for generating reporter A/NY-r19-Gluc virus. A/NY-r19 virus was generated in parallel as control. (**b**) MDCK cells were infected with NY-r19-Gluc virus from serial passage experiments in MDCK cells (passages 1 to 5) at a MOI of 0.01. At 60 h p.i., luciferase assays were performed. (**c**) MDCK cells were mock-infected or infected with NY-r19 and NY-r19-Gluc virus of the indicated passages, respectively, at a MOI of 1. Cytopathic effects were observed at 60 h p.i. using Olympus inverted phase contrast microscope IX73 (magnification 200×).

X-31, which is a 6:2 genetic reassortant encoding the HA and NA genes of A/Hong Kong/1/1968 (H3N2) in a backbone comprising the six internal genes of PR8, is often used in studies requiring an IAV H3N2 subtype [35,36,37,38,39,40,41,42,43]. A similar strategy was explored here, and a reassortant virus NY-r19 encoding HA and NA of the NY virus in the backbone of PR8 was successfully rescued by reverse genetics. In parallel, we further generated a reporter virus NY-r19-Gluc containing HA and NA of the NY virus in the backbone of PR8 where a reporter Gluc gene was fused to NS1 (Figure 3a).

The stability of Gluc in the reassortant virus NY-r19-Gluc was determined by serial passages in MDCK cells. As shown in Figure 3b, no loss of Gluc expression was observed upon virus passaging (for at least 8 passages), suggesting that the reassortant virus is stable *in vitro*. The CPEs in MDCK cells respectively infected with NY-r19-Gluc of early and late passages were also compared, and no difference was detected (Figure 3c)

Together, these results show that reassortant NY-r19-Gluc virus is a good candidate as a reporter influenza A subtype H3N2 virus for further characterization and application.

### 3.4. Characterization of the Reassortant Reporter NY-r19-Gluc Virus in Vitro

**Figure 4:**
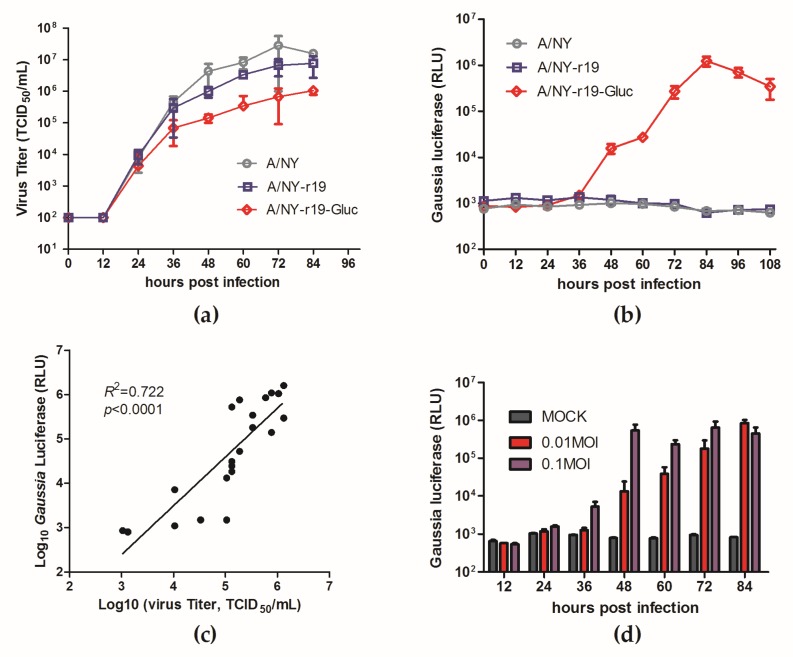
In vitro characterization of NY-r19-Gluc virus. (**a**–**c**) Madin-Darby canine kidney (MDCK) cells were infected with A/NY-r19–Gluc, A/NY-r19, and influenza A virus (IAV) NY at a multiplicity of infection (MOI) of 0.01. At indicated times post-infection, (**a**) viral titers and (**b**) *Gaussia* luciferase activities were respectively determined. (**c**) Correlation between supernatant luminescence and infectious virus titers (*R*^2^ = 0.722, *p* < 0.0001; GraphPad Prism 5, La Jolla, CA, USA). (**d**) *Gaussia* luciferase signals derived from supernatants of NY-r19-Gluc virus-infected cells at MOIs of 0.01 and 0.1, respectively.

To determine the replication properties of the recombinant virus, the growth kinetics of NY-r19-Gluc virus was evaluated and compared to that of wild-type NY and NY-r19. As shown in Figure 4a, NY-r19 showed a growth pattern similar to that of wild-type NY, while NY-r19-Gluc replicated less efficiently, with a titer roughly 15 times lower than the wild-type virus. The expression of Gluc over the virus growth period of NY-r19-Gluc was also detected. Unlike virus titers, the Gluc activity was not detectable until 36 h p.i., suggesting a slight delay in luminescence kinetics (Figure 4b). However, the Gluc level grew rapidly after that and showed a significant correlation with the accumulation of the infectious viruses (Figure 4c). In addition, the luciferase signal correlated well with the MOI of NY-r19-Gluc infecting MDCK cells (Figure 4d).

Collectively, these data demonstrate that the reporter influenza A subtype H3N2 virus NY-r19-Gluc is replication-competent, and viral proliferation can be monitored by the Gluc assay.

### 3.5. Use of NY-r19-Gluc to Test Group-2-Specific HA Inhibitors

Considering that HA is a promising target for antiviral development and given the subtype-specificity of HA inhibitory small molecules, we tested NY-r19-Gluc against some group-2-specific HA inhibitors. To achieve this, MDCK cells were infected with NY-r19-Gluc at a MOI of 0.1, in presence of TBHQ, an inhibitor specifically targeting group-2 HAs, and arbidol, a broad spectrum HA inhibitor, respectively. At 72 h p.i., the expression level of Gluc was determined to monitor virus replication.

**Figure 5:**
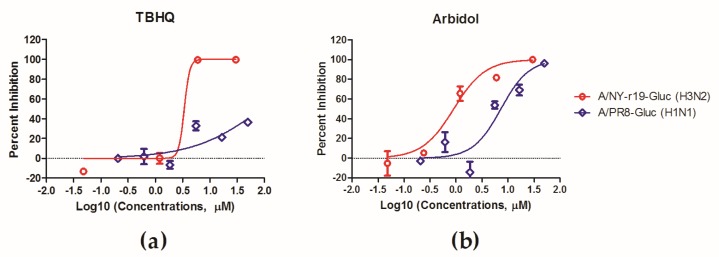
In vitro antiviral determination using NY-r19–Gluc virus as a tool. MDCK cells were infected with the NY-r19–Gluc virus at a MOI of 0.1 in presence of (**a**) TBHQ and (**b**) arbidol, respectively. Infections by PR8-Gluc were performed in parallel as a control. Virus infections were monitored by Gluc examination, and the inhibitory effects were analyzed using GraphPad Prism 5.

As shown in Figure 5, NY-r19-Gluc virus was sensitive to both TBHQ and arbidol, with an IC_50_ of 3.4 μM and 0.9 μM, respectively. In contrast, PR8-Gluc virus, which possesses a group-1 HA, was inhibited by arbidol but not by TBHQ. These results indicate that the NY-r19-Gluc virus can be used to evaluate IAV antivirals.

## 4. Discussion

Reporter viruses provide a powerful tool for basic virology studies as well as antiviral development. However, the introduction of foreign genes or modifications of existing viral genes can lead to altered the virological properties, such as delayed replication kinetics or reduced virulence; moreover, such alterations can create an evolutionary pressure, leading to loss of the reporter genes [44]. In the present study, the strategy of fusing reporter genes to the NS1 open reading frame that was successfully used to construct reporter influenza A subtype H1N1 viruses [28,45] failed to generate a reporter influenza A subtype H3N2 virus (Figure 1). This is not surprising, since our previous study demonstrated that the vRNA replication of an IAV segment can be impaired by the insertion of exogenous genes, resulting in an imbalance of the eight segments during genome replication [33]. We speculate that the NS segment of IAV H3N2 is more vulnerable than that of H1N1 to the modification.

A second strategy using the PR8 NS gene instead of that of the NY virus to express NS1-Gluc chimeric protein was also explored in this study, and a reporter influenza A subtype H3N2 virus NY-r18-Gluc was successfully rescued. This reporter virus can express a high level of Gluc protein, and the reporter gene is adequately maintained upon passaging in MDCK cells (Figure 2b). However, we observed a change in CPE in the MDCK cells infected with NY-r18-Gluc virus after passage 4 (Figure 2c). It is likely that the mutations in the genome of the recombinant virus occurred during the passages, and the mutations are likely residues within the HA gene, since the CPE alteration includes syncytia formation, which is mediated by HA. Thus, this recombinant virus may have limited utility as a research tool.

Using a third strategy, we successfully generated a reporter influenza subtype H3N2 virus NY-r19-Gluc, which is a 6:2 genetic reassortant encoding the HA and NA genes of the NY virus in a backbone comprising the six internal genes of PR8, where a Gluc gene is fused to the NS segment as previously described [28]. Although slightly attenuated compared to wildtype virus, the reassortant virus is replication-competent and stable *in vitro*; furthermore, the expression level of Gluc protein can be used to monitor virus replication accurately (Figure 3 and Figure 4).

The usefulness of the NY-r19-Gluc virus as a tool to evaluate subtype-specific HA inhibitors was validated with TBHQ, an inhibitor specifically targeting group-2 HAs, and arbidol, a broad-spectrum HA inhibitor [21,22,24]. The luciferase assay clearly demonstrated that the NY-r19-Gluc virus is sensitive to both TBHQ and arbidol, with IC_50_ values comparable to the results using traditional methods [21,46,47]. Similarly we think that the NY-r19-Gluc virus can be used to evaluate antibodies or vaccines against IAVs.

In summary, a stable replication-competent reassortant influenza A subtype H3N2 virus carrying a Gluc gene was generated here. This reporter virus can be adapted as a powerful tool, providing simple and robust assays especially for IAV studies of subtype-specificity.

## Figures and Tables

**Table 1:** Primers used to construct pDZ-NY/NS-Gluc.

Primers	Sequences
5′-SDM-NYNS-SAnull-F	5′-CACCATTGCCTTCTTTCCCGGGACATACTATTGAGG-3′
3′-SDM-NYNS-SAnull-R	5′-CCTCAATAGTATGTCCCGGGAAAGAAGGCAATGGTG-3′
5′-INFU-NCR-NYNS1-F	5′-CGACCTCCGAAGTTGGGGGGGAGCAAAAGCAGG-3′
3′-INFU-NYNS1-nostop-R	5′-GGTTGGCATTCCGGACCCAACTTTTGACCTAGCTGTTCT-3′
5′-INFU-Gluc-F	5′-GGGTCCGGAATGCCAACCGAGAACAAC-3′
3′-INFU-Gluc-2A-R	5′-TGACACAGTGTTGGAATCCATCGGGCCCGGGTTTTCTTCCAC-3′
5′-INFU-NYNEP-F	5′-GTGGAAGAAAACCCGGGCCCGATGGATTCCAACACTGTGTCAAGTTTCCAGGACATACTATTGAGGATGTC-3′
3′-INFU-NYNEP-NCR-R	5′-GCATTTTGGGCCGCCGGGTTATTAGTAGAAACAAGG-3′
